# Multi-Omics Profiling Identifies a High-Risk Subgroup of Breast Cancer Stem Cells for Prognostic Stratification and Personalized Treatment

**DOI:** 10.7150/jca.109589

**Published:** 2025-02-28

**Authors:** Guixin Wang, Ziyi Chen, Yao Tian, Yuxin Zhu, Shuo Wang, Wenbin Song, Xin Wang, Yingxi Li

**Affiliations:** 1 The First Department of Breast Cancer, Key Laboratory of Cancer Prevention and Therapy, Tianjin's Clinical Research Center for Cancer, National Clinical Research Center for Cancer, Key Laboratory of Breast Cancer Prevention and Therapy, Tianjin Medical University Cancer Institute and Hospital, Tianjin Medical University, Tianjin, 300060, China.; 2 Department of Thoracic Oncology, Tianjin Lung Cancer Center, Key Laboratory of Cancer Prevention and Therapy, Tianjin's Clinical Research Center for Cancer, Tianjin Medical University Cancer Institute and Hospital, Tianjin Medical University, Tianjin, 300060, China.; 3 Department of General Surgery, Tianjin Medical University General Hospital, Tianjin Key Laboratory of Precise Vascular Reconstruction and Organ Function Repair, Tianjin General Surgery Institute, Tianjin, 300052, China.; 4 Tianjin Medical University, Tianjin, 300070, China.; 5 Immunology Department, Key Laboratory of Immune Microenvironment and Disease (Ministry of Education), Tianjin Medical University, Tianjin, 300070, China.

**Keywords:** breast cancer, cancer stem cells, heterogeneity, multi-omics, tumor microenvironment

## Abstract

**Background:** Breast cancer is the most prevalent malignancy among females worldwide. Extensive research has highlighted cancer stem cells (CSCs) as critical drivers of tumor initiation, progression, recurrence, and therapy resistance. However, the heterogeneity of breast cancer stem cells (BCSCs) and their dynamic roles within the tumor microenvironment remain inadequately understood.

**Methods:** This study utilized the single-cell RNA sequencing dataset to categorize BCSCs into two subgroups within the breast cancer microenvironment and investigate their pseudo-time developmental dynamics. Bulk transcriptomic data from TCGA-BRCA were integrated to assess the prognostic significance and infiltration abundance of the BCSCs-2 subgroup. Functional enrichment, co-expression network analysis, and somatic mutation profiling were performed to elucidate key biological pathways and genetic features. Additionally, drug sensitivity analyses were conducted using the Connectivity Map database to identify potential therapeutic strategies.

**Results:** A total of 459 BCSCs were identified and further classified into two distinct subpopulations: BCSCs-1 and BCSCs-2. High infiltration of BCSCs-2 was associated with poor prognosis and an immunosuppressive tumor microenvironment. Co-expression network analysis identified 16 key genes linked to BCSCs-2, while somatic mutation analysis revealed distinct mutation patterns associated with its infiltration. Drug sensitivity analysis suggested that patients with high BCSCs-2 infiltration could benefit from classical chemotherapy agents, such as Cisplatin, and other novel therapeutic compounds.

**Conclusions:** This study offers novel insights into the heterogeneity and functional roles of BCSCs in breast cancer. The findings highlight the prognostic and therapeutic importance of the BCSCs-2 subgroup, providing potential biomarkers and therapeutic targets for precision medicine in breast cancer management.

## Introduction

With the advancement of medical technology and the increase in human life expectancy, cancer has emerged as a significant global public health concern^1^, ^2^. Notably, breast cancer poses a particularly severe threat to female health worldwide, accounting for one-quarter of all newly diagnosed cancer cases and one-sixth of cancer-related deaths among females globally^3^. Although breast cancer mortality rates have declined in some countries, its incidence continues to rise, increasing by 1% annually from 2012 to 2021^4^. Molecular subtyping of breast cancer has become a widely accepted approach for guiding therapy in breast cancer patients^5^. However, accumulating evidence indicates that molecular subtyping cannot completely capture the heterogeneity of breast cancer. Therefore, it is urgent to deeply explore the tumor microenvironment to address this challenge.

Cancer stem cells (CSCs) are a subset of tumor cells that exhibit stem cell-like characteristics and have been recognized as key players in the initiation and progression of tumors^6^. These cells form a rare and highly organized hierarchical structure within the tumor and are considered the root cause of cancer recurrence and resistance to treatment. As such, CSCs have been a central focus of cancer research^7^. Cancer stem cells possess traits such as tumor initiation, self-renewal, multipotency, plasticity, and cancer stemness as a cellular state. These characteristics not only contribute to tumor progression and dissemination but also indicate that CSCs are in a dynamic and variable state within the tumor microenvironment^7^. Clinically, treatments targeting cancer stem cells have been tested in some blood cancers and solid tumors^6^. In lung cancer, Notch activity has been shown to identify cancer stem-like populations, and its inhibition may serve as a potential target for treating lung adenocarcinoma^8^. In summary, increasing evidence suggests that many cancers are driven by CSCs, which possess self-renewal and unlimited differentiation potential. Therefore, research on CSCs is crucial for the development of more effective cancer therapies.

Breast cancer stem cells (BCSCs) were first identified in 2003 using a model in which human breast cancer cells grew in immunodeficient mice^9^, ^10^. With deeper investigation into BCSCs, it has been found that they are divided into several distinct subpopulations, each with different functions^11^, ^12^. At the same time, potential therapeutic strategies targeting BCSCs have been reported. For example, treatment with bevacizumab, combined with the dual-topoisomerase inhibitor TOPO-1 and the HIF-1α inhibitor camptothecin, has shown potential for effectively targeting CSCs under hypoxic conditions^13^. Additionally, some reports indicate that CSC-targeting drugs can significantly enhance the effectiveness of anti-angiogenic therapies by targeting key pathways involved in tumor growth and vascularization^14^. Meanwhile, the plasticity of BCSCs has attracted significant research attention^15^. This suggests that BCSCs may play a key role in breast cancer treatment and hold substantial clinical translational value. However, our understanding of BCSCs is still in its early stages. Given the dynamic nature of BCSCs within the tumor microenvironment, it is essential to identify their subpopulations.

Although previous studies have suggested that targeting CSCs may enhance the efficacy of traditional cancer treatments, the identification and eradication of CSCs remain challenging^16^. With the development and maturation of single-cell sequencing technology, we are now able to more reliably identify CSCs within the tumor microenvironment^17^, ^18^. Therefore, this study aims to utilize single-cell RNA sequencing to identify CSCs in breast cancer and classify them into different subpopulations. We explored the developmental trajectory of different BCSCs and their association with patient prognosis. Furthermore, we investigated the roles and functions of these subpopulations within the tumor microenvironment. We also identified key cellular markers and co-mutation patterns of the newly discovered BCSCs. Notably, drug sensitivity analysis further highlighted the clinical translational value of our research. Overall, this study provides a new perspective for understanding breast cancer from the viewpoint of CSCs and offers a novel potential therapeutic strategy for breast cancer treatment.

## Materials and Methods

### Data Acquisition and Pre-processing

Bulk-RNA transcriptome data, single nucleotide variant (SNV) data, and survival time-to-event data of corresponding patients were obtained from the BRCA cohort in The Cancer Genome Atlas (TCGA). The 'TCGAbiolinks' package (v2.26.0) was used to obtain the above data. Patients were included based on following criteria: 1. Complete expression profiles and follow-up information; 2. Diagnosed with primary breast cancer. In summary, a total of 1080 breast cancer patients with bulk RNA data were enrolled in study. The single-cell RNA sequencing dataset (GSE180286, https://www.ncbi.nlm.nih.gov/geo/query/acc.cgi?acc=GSE180286) was obtained from the Gene Expression Omnibus (GEO) Series. Of note, 5 single-cell profiles of primary sites were included for subsequent analyses.

The Seurat (v4.3.0) package was used for quality control and dimensionality reduction of scRNA-seq profiles. Routine quality control (QC) was performed using the following threshold criteria: genes detected per cell ≥ 200; nCount of RNA ≥ 200; proportion of reads mapped to mitochondrial genes ≤ 20%; proportion of reads mapped to hemoglobin genes ≤ 1%; and log10FeaturePerUMI ≥ 0.8. Potential doublets and batch effects were removed using the DoubletFinder package (v2.0.3)^19^ and Harmony (v1.2.0)^20^, respectively. The number of low-quality cells removed for each sample to 0.8% of the total cell count. SCTransform (v0.3.5)^21^ was conducted for normalization, variance stabilization, and scaling of scRNA-seq data. Cell population annotation was performed based on biological markers obtained from online database^22^.

### Downstream Analysis of scRNA-seq Data

The SCP (v0.4.7.9) package was used to conduct pseudo-time analysis to simulate the developmental trajectory of cells and identify key markers, functional annotations, and pathway analysis among different cell clusters. Specifically, the Slingshot tool was used to calculate the similarity and distance between cells, thereby inferring the dynamic trajectories of cells in a specific biological process within a low-dimensional space. The single-cell matrix and bulk transcriptome data were integrated using CIBERSORTx (https://cibersortx.stanford.edu/)^23^ to estimate the infiltration abundance of BCSCs-2 in different samples. The single-cell gene matrix of BCSCs was used as the reference, while the bulk RNA matrix was utilized for inference. The parameters for the single-cell input options were set as follows: 1. min.Expression = 0.75; 2. Replicates = 5; 3. Sampling = 0.5. Additionally, the absolute mode was selected to infer the abundance of cell clusters in the sample tissues. Subsequently, the survival outcomes of different groups based on the best cut-off value was evaluated using the survminer (v0.4.9) R package through Kaplan-Meier analysis.

### Enrichment Analysis

Differential gene expression analysis was performed using the limma (v3.54.2) package. Differentially expressed genes were identified based on the P-value, with P < 0.05 considered significant. All differential genes were reordered by the value of fold change. Subsequently, gene set enrichment analysis (GSEA) provided by the clusterProfiler (v4.12.2) R package^24^ was used to further identify potential functional pathways associated with BCSCs-2, and the GseaVis (v0.0.5) package was used for visualization.

### Co-expression Network Analysis

The "hdwgcna" (v0.2.18) R package was used to analyze co-expressed gene modules in breast cancer-associated stem cells. Weighted Gene Co-expression Network Analysis (WGCNA) is a commonly used method for gene expression data analysis that identifies gene modules and investigates the association between these modules and external sample characteristics. Specifically, based on the expression profile variation of BCSCs, we chose 12 as the soft threshold and divided them into 13 different modules. The FindMarkers function in the Seurat package was used to identify the top genes of the BCSC-2 cell population, and the ggvenn (v0.1.10) package was used to perform an intersection and obtain the list of genes of interest.

### Somatic Mutation Landscape Analysis

The TCGAbiolinks package (v2.26.0) was used to access the required somatic mutation data from the TCGA database. The landscape of masked somatic mutation profiles between different BCSCs-2 infiltration abundances was analyzed using the maftools (v2.20.0) package^25^. The significance of gene pairs between different groups was identified using Fisher's exact test. Additionally, a heatmap was generated to illustrate the co-occurrence and mutual exclusivity of mutations across genes.

### Drug Sensitivity Analysis

Connectivity Map (CMap, https://clue.io/)^26^ was used to further explore potential corresponding drugs by comparing gene expression profiles. CMap is a reliable bioinformatics database and tool that can identify candidate compounds capable of reversing specific disease-associated gene expression patterns, thereby providing insights for novel drug development. The ggplot2 (v3.5.1) package and ComplexHeatmap (v2.16.0) package were used for the visualization of CMap results.

In addition, the oncoPredict (v1.2) package was used to analyze the drug sensitivity of several chemotherapeutics between distinct groups.

### Statistics

All statistical methods were performed using R software (version 4.2.2). Specific statistical details can be found in the corresponding material and methods section.

## Results

### Identification of Breast Cancer Stem Cells

**Figure 1:**
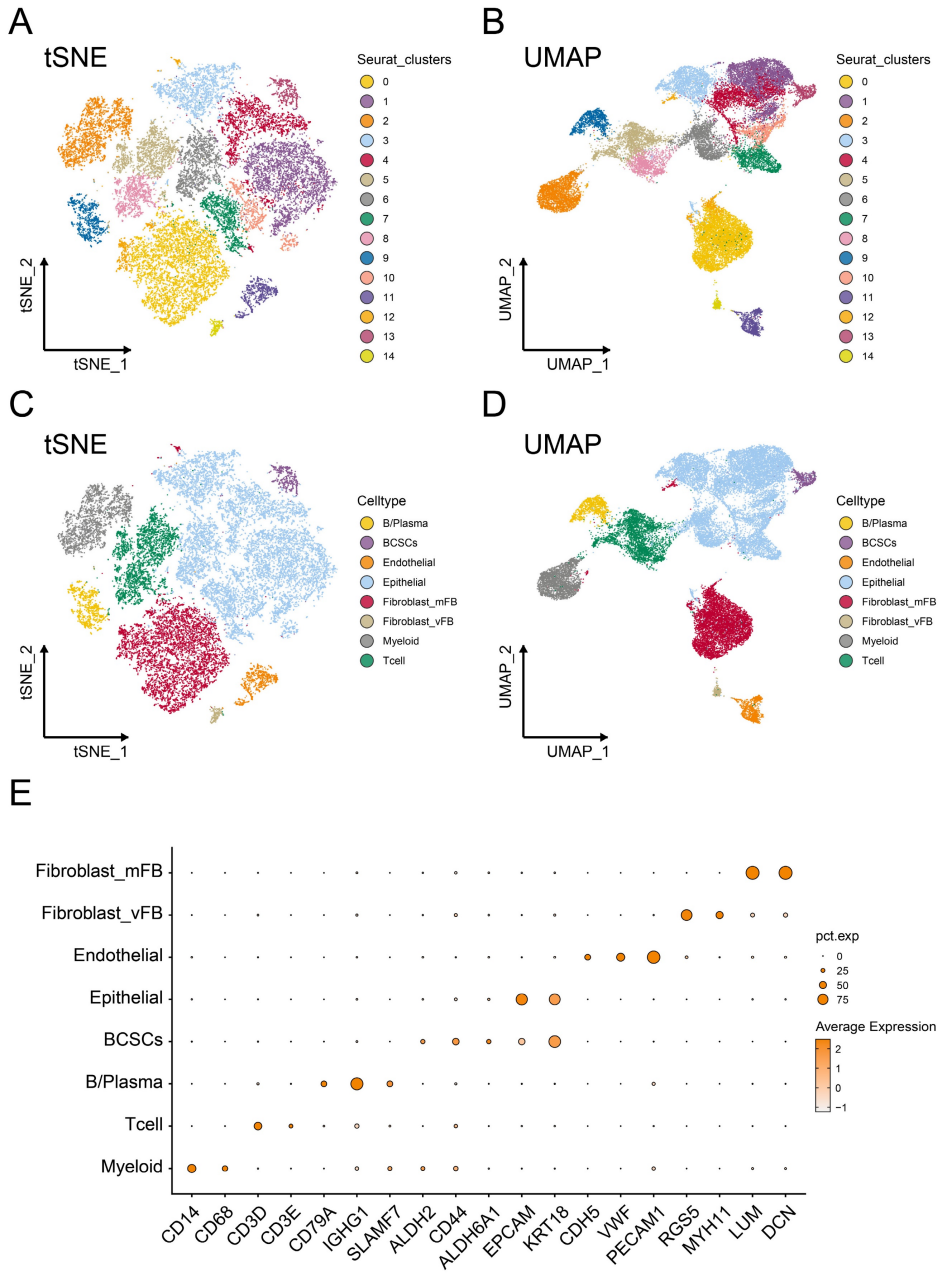
** Tracking and annotating breast cancer stem cells from single-cell transcriptome data.** (**A-B**) Reduced dimension maps of t-Distributed Neighbor Embedding (t-SNE) and the Uniform Manifold Approximation and Projection (UMAP) based on clusters. (**C-D**) Reduced dimension maps of t-Distributed Neighbor Embedding (t-SNE) and the Uniform Manifold Approximation and Projection (UMAP) based on cell types. (**E**) The expression levels of cell marker genes in different cell population annotations were represented by dot plots.

As illustrated in Figure 1, this study aims to investigate the presence of breast cancer stem cells in the breast cancer microenvironment and explore their potential biological functions. After the standardized quality control process of the single-cell RNA transcript profile, potential low-quality doublets were removed to ensure the reliability of the data used for subsequent analysis.

Subsequently, we applied t-Distributed Neighbor Embedding (t-SNE) (Figure 1A) and Uniform Manifold Approximation and Projection (UMAP) (Figure 1B) methods for dimensionality reduction, resulting in the partitioning of the 25,665 cells into 14 distinct clusters with the resolution set as 0.5. Furthermore, based on previously reported biologically specific markers of different cell populations, we constructed the single-cell profile and identified 459 breast cancer stem cells (BCSCs), 949 B cells/plasma cells, 792 endothelial cells, 12173 epithelial cells, 2385 myeloid cells, 3074 T cells, 5632 myofibroblasts, and 201 vascular fibroblasts (Figure 1C-D).

It is worth noting that CD14 and CD68 were used to label myeloid cells, while CD3D and CD3E served as markers for T cells. CD79A, IGHG1, and SLAMF7 were utilized to identify B cells and plasma cells. EPCAM and KRT18 were employed to label epithelial cells, whereas ALDH2, CD55, and ALDH6A1 were used to identify breast cancer stem cells. CDH5, VWF, and PECAM1 were markers for endothelial cells, and RGS5, MYH11, LUM, and DCN were used to label fibroblasts (Figure 1E).

### Cell Trajectory of Breast Cancer Stem Cells

**Figure 2:**
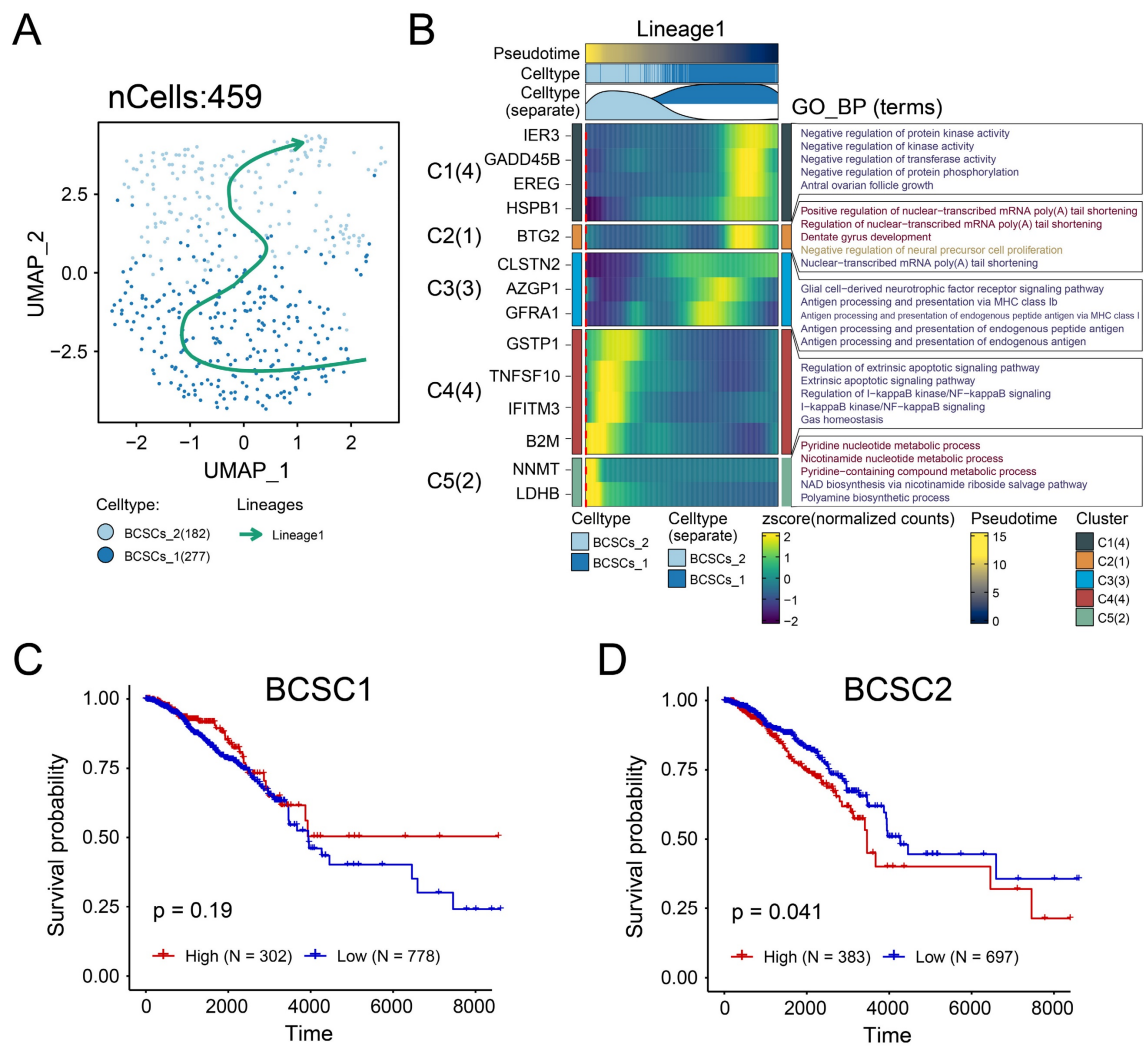
** Developmental trajectory and prognostic analysis of BCSCs.** (**A**) Pseudo-time developmental trajectories of BCSCs. BCSCs tended to develop from BCSCs-1 to BCSCs-2. (**B**) Changes in gene expression of BCSCs in Lineage 1, with functional enrichment results of GO_BP for different gene lists shown on the right. (**C**) Kaplan-Meier curves describing overall survival (OS) in breast cancer patients with different infiltration levels of BCSC-1. (**D**) Kaplan-Meier curves describing overall survival (OS) in breast cancer patients with different infiltration levels of BCSC-2.

Cells in the tumor microenvironment are constantly undergoing dynamic changes. Therefore, to further explore the heterogeneity of breast cancer stem cells in the tumor microenvironment, we conducted pseudo-time analysis. Two distinct subgroups and a potential lineage evolution of breast cancer stem cells were identified. BCSCs showed a tendency to develop from BCSCs-1 to BCSCs-2 (Figure 2A). The top 5 markers of each cell subgroup were illustrated in supplementary Figure S1A. In order to further study the potential biological functions of BCSCs, the changes in gene expression between different cell populations and pseudo-time stages were examined. The functional context of the biological processes associated with these genes was also investigated through Gene Ontology Biological Process (GO_BP) terms (Figure 2B).

Notably, during the developmental trajectory of cells, breast cancer stem cells (BCSCs) tend to express genes such as IER3 and GADD45B at the early stage, while genes like NNMT and LDHB are preferentially expressed in the BCSCs-2 developmental state. In terms of their biological functions, early-stage BCSCs-1 cells could regulate stress responses and signal transduction by inhibiting protein kinase activity through pathways like 'Negative regulation of protein kinase activity' and 'Negative regulation of protein phosphorylation'. The enrichment of BCSCs-2 cells in the 'Pyridine nucleotide metabolic process' and 'NAD biosynthetic process' suggested a metabolically active phenotype. These results suggest that the intrinsic lineage evolution of BCSCs may be accompanied by the evolution of distinct biological functions.

### Tumor Prognostic Value and Potential Biological Role of BCSCs-2 Cells

In order to reveal the clinical significance of breast cancer stem cells, we used the sc-RNA matrix to estimate the abundance of stem cell infiltration levels of patients in the TCGA-BRCA cohort. The high- and low- infiltration groups were determined by the best cut-off value based on the absolute score of each cluster. Interestingly, a high abundance level of BCSCs-1was found to have no significant prognostic value (Figure 2C), while a high abundance level of BCSCs-2 was associated with poor prognosis (p < 0.05, Figure 2D). Based on these findings, we investigated whether BCSCs-2 might influence prognosis through specific biological functions.

**Figure 3:**
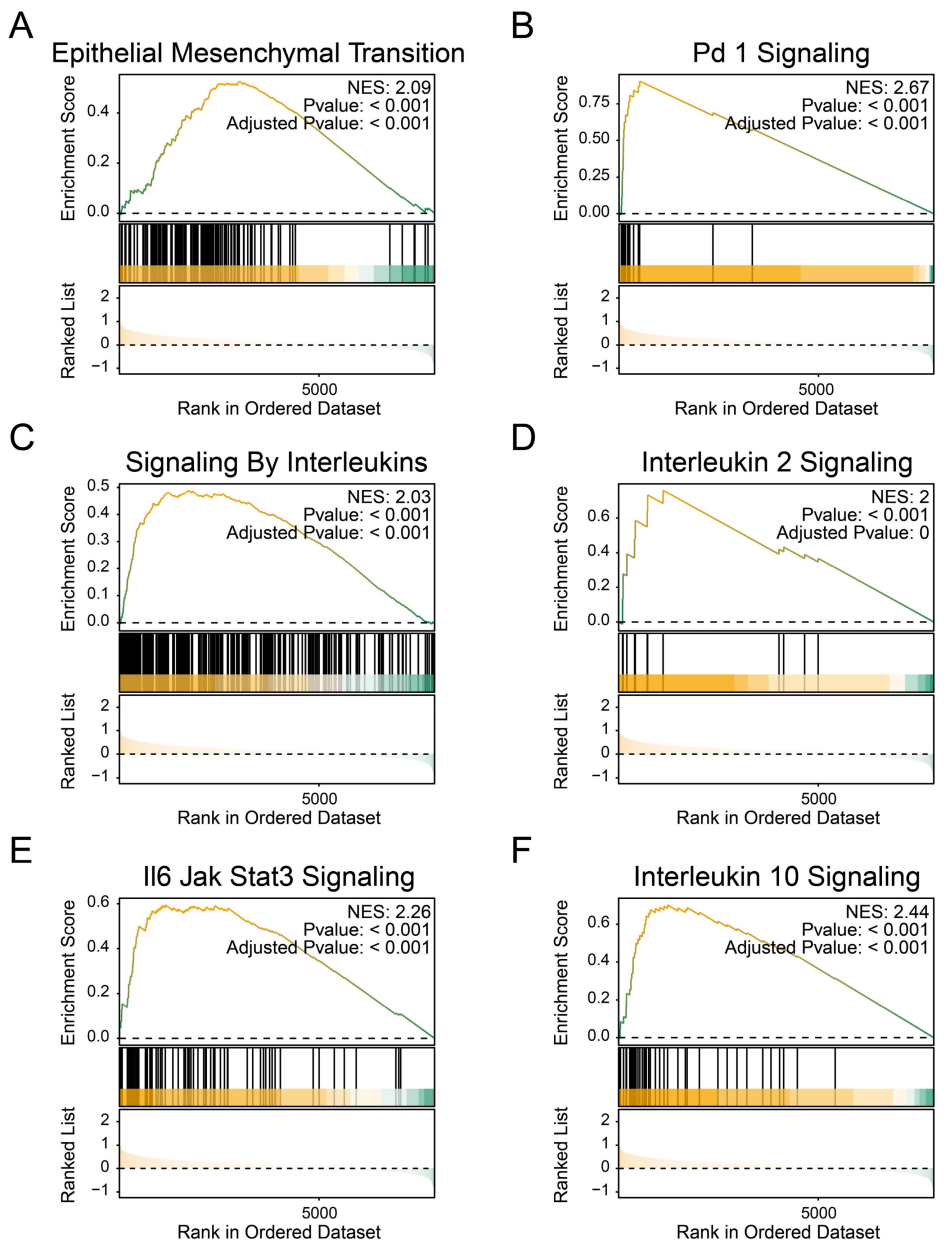
** The enrichment of BCSCs-2 may reflect an increase in tumor malignancy. A-F** The GSEA plot based on the signature gene sets reveals significant enrichment of different biological pathways in patients with high BCSCs-2 cell infiltration abundance. "Epithelial Mesenchymal Transition" (**A**), "PD-1 Signaling" (**B**), "Signaling By Interleukins" (NES: 2.03, Adjusted p-value: < 0.001, **C**), "Interleukin 2 Signaling" (**D**), "IL-6 JAK/STAT3 Signaling" (**E**), and "Interleukin 10 Signaling" (**F**).

The transcriptome data were used to obtain the list of differentially expressed genes, which were sorted by fold change and analyzed by GSEA functional enrichment. We observed a downregulation of the following pathways in the high BCSCs-2 group: 'Epithelial Mesenchymal Transition' (NES: 2.09, Adjusted p-value: < 0.001, Figure 3A), 'PD-1 Signaling' (NES: 2.67, Adjusted p-value: < 0.001, Figure 3B), 'Signaling By Interleukins' (NES: 2.03, Adjusted p-value: < 0.001, Figure 3C), 'Interleukin 2 Signaling' (NES: 2.00, Adjusted p-value: < 0.001, Figure 3D, Supplementary Table S1), 'IL-6 JAK/STAT3 Signaling' (NES: 2.26, Adjusted p-value: < 0.001, Figure 3E), and 'Interleukin 10 Signaling' (NES: 2.44, Adjusted p-value: < 0.001, Figure 3F). These findings indicate that the high infiltration level of BCSCs-2 may be associated with heightened tumor malignancy, enhanced tumor stemness, and the formation of an immunosuppressive microenvironment.

### Identification of Gene Co-expression Modules Among BCSCs

**Figure 4:**
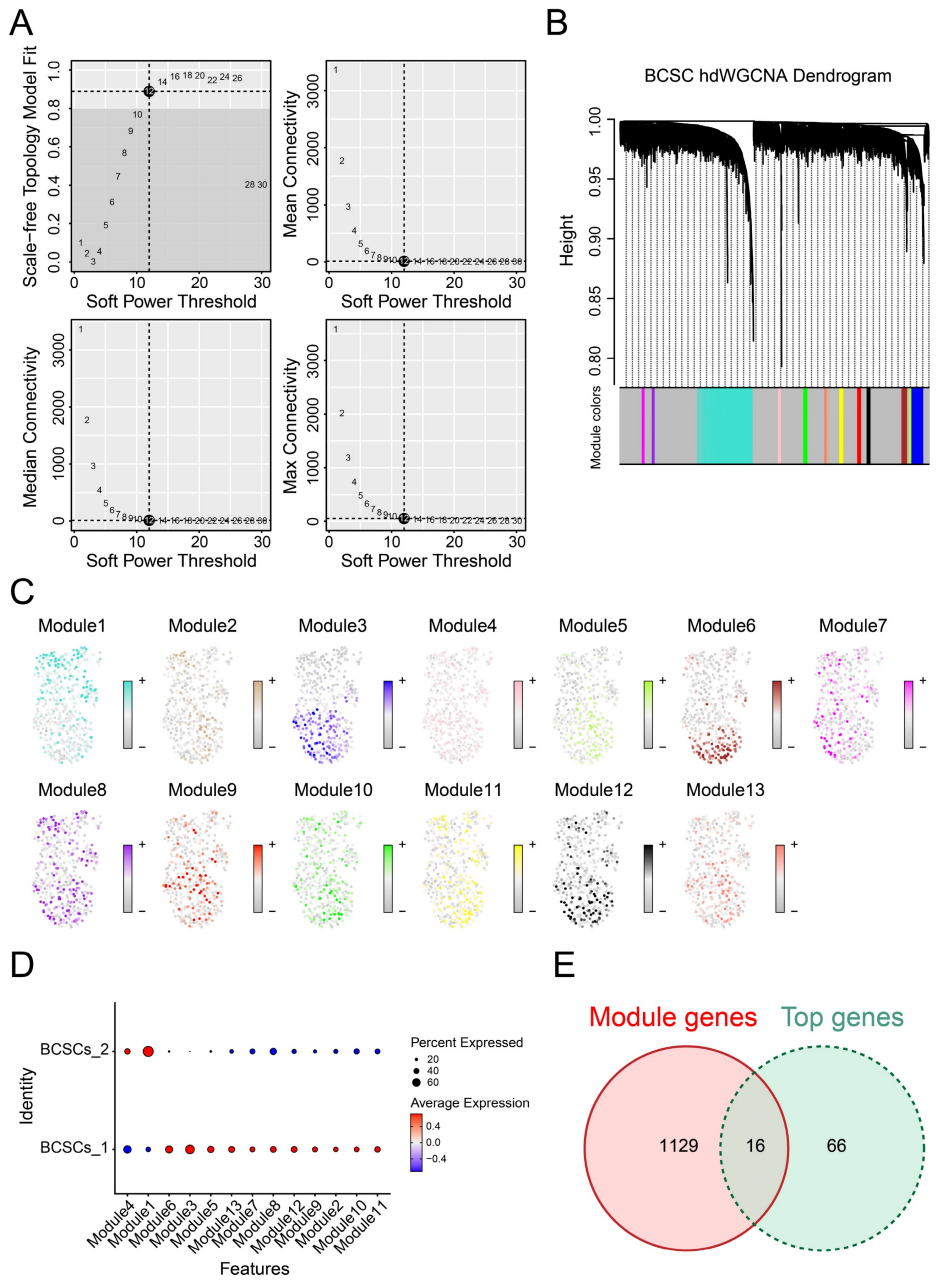
** Biological markers of BCSCs-2 were identified with gene co-expression modules.** (**A**) Weighed gene co-expression network analysis was constructed among BCSCs. (**B**) The hdWGCNA dendrogram of 13 modules. (**C**) The distribution of characteristic genes of each module. (**D**) Dotplot was used to show the expression of characteristic genes in different modules. (**E**) Intersection of module genes and BCSCs differentially expressed genes.

Due to the correlation between breast cancer stem cell subpopulations and prognosis as well as biological functions, it is essential to explore the co-expression gene networks that play significant roles in these two subgroups. To achieve optimal connectivity, we constructed a scale-free network for BCSCs and set the soft threshold to 12 (Figure 4A-B). Finally, we identified a total of 13 modules and found that the genes in module 1 were most enriched and played a critical role in BCSCs-2, while they were scarcely enriched in BCSCs-1(Figure 4C-D), suggesting these genes could be important co-expressed genes. In addition, we performed differential gene analysis between BCSCs-2 and other types of cells to obtain the top marker genes of BCSCs-2. By integrating the above gene lists, we identified 16 intersecting genes (Figure 4E), including KMT2E, IFITM3, MAGED2, TM4FSF1, GSTP1, SRI, CD59, SLC39A6, LIMCH1, ATP1B1, DSP, SYNE2, RARRES3, TMC5, SLC40A1, and TM4SF18. In summary, the above genes may serve as potential diagnostic markers and core genes of BCSCs-2.

### BCSCs-2 Cells Possessed More Somatic Co-mutations

**Figure 5:**
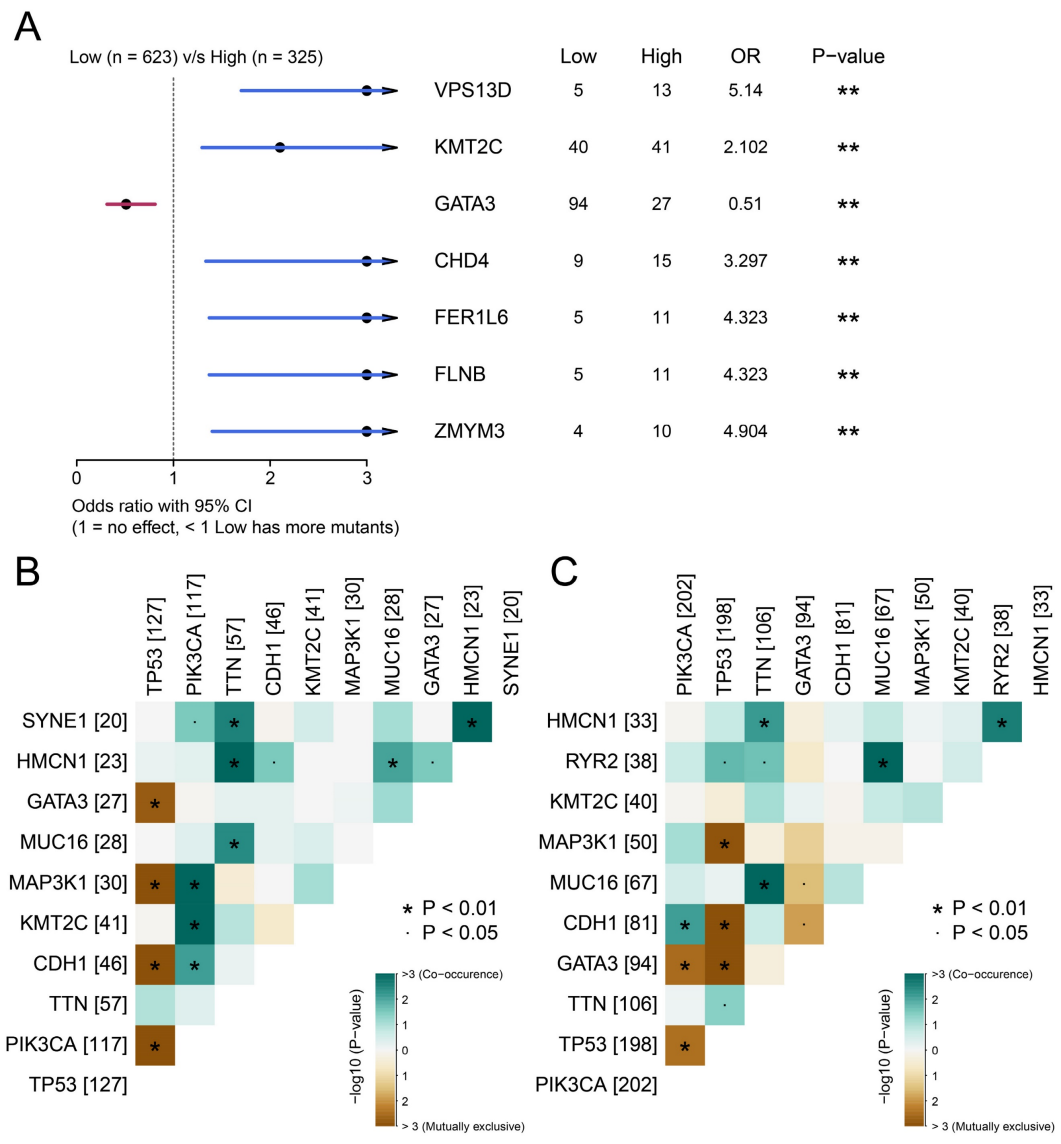
** Associates between the infiltration abundance of BSCSs_2 and somatic mutations.** (**A**) The forest plot illustrates the differences in somatic single-nucleotide variants (SNVs) between patients with high and low BCSCs-2 cell infiltration. The OR > 1 indicates a higher SNV frequency in the high BCSCs-2 infiltration group, the OR < 1 indicates a higher SNV frequency in the low infiltration group, and the OR = 1 suggests similar SNV frequencies between the two groups. (**B**) Landscape of gene co-mutations with high infiltration of BCSCs-2. (**C**) Landscape of gene co-mutations with low infiltration of BCSCs-2.

The above results confirmed the prognostic value of BCSCs-2, and we next explored the factors that may relate to the infiltration level of BCSCs. After integrating SNV data and removing invalid entries, we further analyzed the significant differences in somatic mutations between the two groups using Fisher's exact test. Genes such as VPS13D (p < 0.01), KMT2C (p < 0.01), CHD4 (p < 0.01), FER1L6 (p < 0.01), FLNB (p < 0.01), and ZMYM4 (p < 0.01) possessed more mutation frequencies in the high BCSCs-2 infiltration group. Meanwhile, GATA3 (p < 0.01) was found to possess more mutations in the low infiltration group (Figure 5A).

Co-mutation patterns were more abundant in the high BCSCs-2 infiltration abundance group than in the other group (Figure 5B-5C). These findings indicated significant differences in the somatic mutation spectrum between the two groups and revealed a subtle link between somatic variation and BCSCs-2.

### Drug Sensitivity Prediction Value of BCSCs-2 Cells

**Figure 6:**
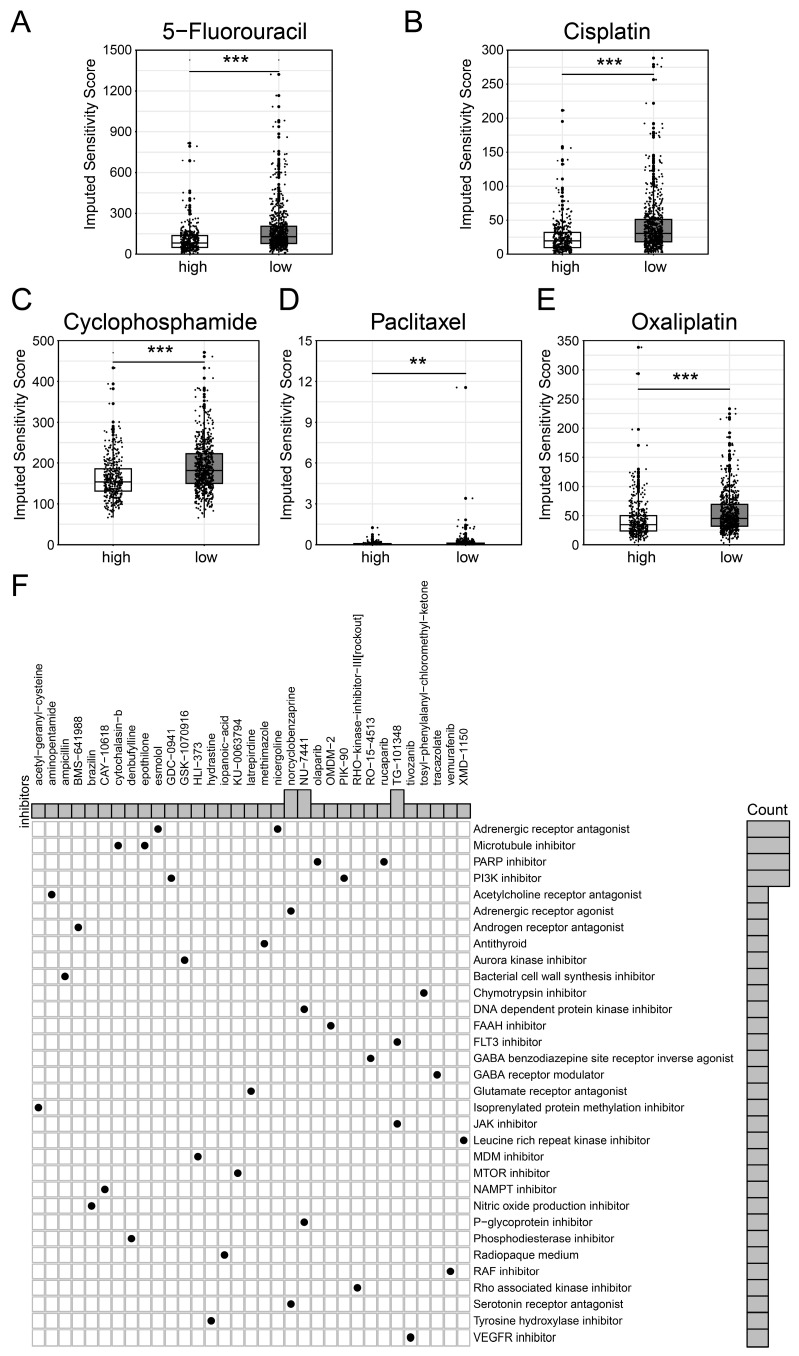
** Differential drug sensitivity analysis for patients with high and low BCSCs_2 cell infiltration.** Boxplots (**A-E**) display the IC50 for standard chemotherapeutic agents, including 5-Fluorouracil (**A**), Cisplatin (**B**), Cyclophosphamide (**C**), Paclitaxel (**D**), and Oxaliplatin (**E**). Statistical significance is denoted by ** (p < 0.01) and *** (p < 0.001). (**F**) Dot plot of inhibitor functional enrichment illustrates the enrichment of inhibitors targeting diverse pathways based on CMAP analysis, with the x-axis representing specific inhibitors and the y-axis indicating their functional categories.

We first employed the 'oncopredict' package to evaluate the IC50 value of several chemotherapy drugs among BCSCs-2 groups. Specifically, patients with high infiltration abundance of BCSCs-2 may exhibit greater sensitivity to drugs such as 5-Fluorouracil (p < 0.001), Cisplatin (p < 0.001), Cyclophosphamide (p < 0.001), Paclitaxel (p < 0.01), and Oxaliplatin (p < 0.001) (Figure 6A-E). Most of these drugs are classic and reliable chemotherapy drugs for breast cancer^27^, ^28^. These findings provide new insights into the use of chemotherapy in breast cancer patients.

In addition, we used the CMAP database to screen potential therapeutic drugs targeting high BCSCs-2 group of patients (Supplementary Table S2). According to the mechanisms of various drugs, medications including norcyclobenzaprine, NU-7411, and TG-101348 had the potential to become specific therapeutic options for patients with high BCSCs-2 infiltration via the 'Adrenergic receptor agonist' pathway, the 'DNA-dependent protein kinase inhibitor' pathway, and the 'FLT3 inhibitor' pathway, respectively (Figure 6F). In conclusion, our study provides new insights and strategies for individualized precision treatment in some breast cancer patients.

## Discussion

As far as we know, this study represents an innovative investigation of the unfavorable role of human BCSCs in tumor microenvironment of breast cancer by integration of multiple-scale data, including genomic data, expression profiles, and single-cell RNA profiles. Notably, we identified novel potential diagnostic biomarkers and therapy targets for BCSCs.

In general, BCSCs have been confirmed to play a critical role in tumor origin, development, recurrence, metastasis and therapy resistance in breast cancer^29^, ^30^. However, the constraints of previous sequencing technologies impede a comprehensive and nuanced characterization of BCSCs heterogeneity at the single-cell level within the intricate context of the tumor microenvironment. Fortunately, the advent of single-cell RNA sequencing and spatial transcriptomics has significantly advanced the ability to address these limitations. A large number of studies confirmed the malignant behavior and the heterogeneity of BCSCs. For instance, Nakayama *et al.*^31^ found the two subtypes of BCSCs (HMGA1-high, CD44/MYC-high) existed in TNBC patients as well as TNBC xenograft models, indicating the heterogeneity and diversity of BCSCs by scRNA-seq and *in vivo* experiments. Ji *et al.*^32^ revealed that BCSCs could promote immune escape in TNBC microenvironment through the upregulation of CTLA4-related signaling of CD8+ T cells.

In addition, Li *et al.*^33^ unveiled a subset of BCSCs with high expression of RAC2 and PTTG1 were significantly enriched in lymph mode metastasis, which might promote the tumor metastasis through modulation of immune system and activation of specific pathways. These studies highlight the interaction of BCSCs in the spatial heterogeneity and tumor microenvironment of breast cancer. Consistently, our study identified a subgroup of BCSCs (BCSCs-2) were associated with poor prognosis in breast cancer. Therefore, we focused on exploring the potential biological functions of BCSCs-2. The immune-related pathways including PD-1, IL-6/ JAK/STAT3, IL-10 signaling were significantly enriched in patients with high infiltration of BCSCs-2. These pathways have been confirmed to immune suppression^34^-^36^. The epithelial-mesenchymal translation was also highly upregulated in high infiltration group. Taken together, our findings revealed the BCSCs-2 may promote immune suppression and tumor metastasis in breast cancer microenvironment, which provides a theory basis on prognostic stratification and therapy targets for breast cancer.

As a high heterogeneous cells, BCSCs present a high plasticity^37^. Exploring the potential cell evolution trajectory of BCSCs can provide insight into the changes in their biological functions. We noticed the translational tendency from BCSCs-1 to BCSCs-2 with the dynamic enrichment of NF-kappaB signaling. Interestingly, NF-kappaB was proved to be involved in BCSC fate and expansion^38^. Also, some metabolic pathways (pyridine nucleotide, nicotinamide nucleotide, and polyamine) were gradually upregulated during the evolution, which highlighted the critical role of metabolic process for the evolution of BCSCs. Given the high risk of BCSCs-2, further identification of its biomarkers is warranted to guide therapy and risk stratification. We identified 16 candidate biomarkers (KMT2E, IFITM3, MAGED2, TM4FSF1, GSTP1, SRI, CD59, SLC39A6, LIMCH1, ATP1B1, DSP, SYNE2, RARRES3, TMC5, SLC40A1, and TM4SF18) for BCSCs-2. Although the efficiency of these biomarkers needs to be validated in clinic. Previous reports have demonstrated their potential. For instance, Singh *et al.*^39^ developed a nanomedicine which could modulate GSTP1 to inhibit glycolysis in BCSCs, resulting in the overall tumor regression of TNBC. In a word, the above gene could be potential diagnostic biomarker and promising targets for breast cancer.

We then analyzed the association between somatic mutations and BCSCs-2 infiltration to better understand the underlying mechanisms of BCSCs-2 infiltration heterogeneity. It was noticed that high abundance of BCSC-2 were associated with somatic mutations of VPS13D, KMT2C, CHD4, FER1L6, FLNB, ZMTM3, while low abundance of BCSCs-2 was closely related to GATA3 mutations. Jiang *et al.*^40^ reported GATA3 mutations were correlated with improved overall survival in breast cancer patients, and were mainly occur in patients with luminal-like breast cancer. Their conclusions support our findings that low-infiltrating BCSCs-2 was associated with a favorable prognosis. In addition, KMT2C/D are most frequently mutated histone methyltransferases and play a tumor-suppressive role in breast oncogenesis^41^. These findings have innovatively revealed the subtle relationship between somatic mutation and BCSCs infiltration heterogeneity, providing a new perspective and theoretical basis for further understanding the heterogeneity of BCSCs in the breast cancer microenvironment. Finally, we identified serval chemotherapy drugs and other potential drug for targeting BCSCs-2.

All in all, our study revealed the prognostic value, intrinsic lineage, potential biological processes, infiltration heterogeneity, personalized therapy strategy of BCSCs. However, the study also has some limitations. For instance, although we employed multi-scale data to explore the role of BCSCs, all the results were based on in silico analyses. The *in vivo* and *in vitro* experiments need to be performed in further study to validate our results. Furthermore, the sample size included in our study is limited. Therefore, we will collect more and different data sets to prove the generality of our conclusions. The bioinformatics is the first step of our study, and we meet the challenges in explaining the molecular mechanisms of our findings now. With a large number of high-quality clinical samples, sequencing profiles, and subsequent experiments, this issue will be addressed.

## Supplementary Material

Supplementary figure and tables.

## Abbreviations

TCGA: The Cancer Genome Atlas Program
GEO: Gene Expression Omnibus
TNBC: Triple-negative breast cancer
BCSCs: breast cancer stem cells
CSCs: Cancer stem cells
SNV: single nucleotide variant
QC: quality control
scRNA-seq: single-cell RNA sequencing
Cmap: Connectivity Map
UMAP: Uniform Manifold Approximation and Projection
tSNE: t-Distributed Neighbor Embedding
OS: overall survival
