# Supplementary material for: Multi-Omics Profiling Identifies a High-Risk Subgroup of Breast Cancer Stem Cells for Prognostic Stratification and Personalized Treatment
Source: J Cancer. 2025 Feb 28;16(6):1860–72. doi: 10.7150/jca.109589 (PMC11905410; doi:10.7150/jca.109589)
Supplement: Supplementary file 1 — Supplementary figure and tables. [file jcav16p1860s1.pdf]

Supplementary Figure

Figure S1

A

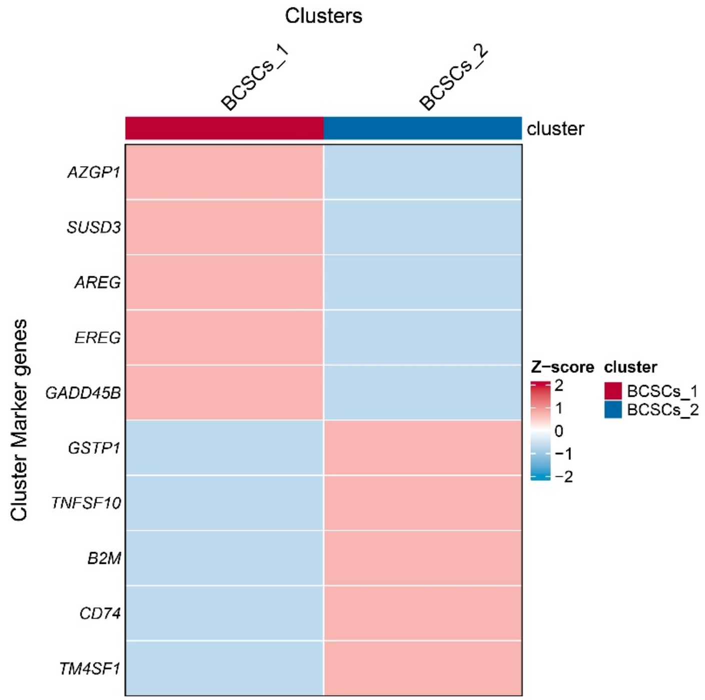

Figure S1 The top represented genes of BCSCs subgroups. **A** Heatmap showing the top 5 markers of each cell group.

Table S1. The GSEA analysis based on IL2-pathway										
ID	Description	setSize	enrichmentScore	NES	pvalue	p.adjust	qvalue	rank	leading_edge	core_enrichment
**REACTOME_INTERLEUKIN_2_SIGNALING**	REACTOME_INTERLEUKIN_2_SIGNALING	12	0.759773833	2.031914912	8.74E-05	0.000843521	0.000602843	1115	tags=67%, list=14%, signal=57%	IL2RB/LCK/IL2RG/JAK3/IL2RA/PTK2B/STAT5A/SYK

Table S2. The candidate drugs for high infiltraion level of BCSCs-2 patients		
Score	Name	MoA
**-94.38**	hydrastine	Tyrosine hydroxylase inhibitor
**-91.91**	methimazole	Antithyroid
**-87.89**	vemurafenib	RAF inhibitor
**-86.21**	NU-7441	DNA dependent protein kinase inhibitor, P-glycoprotein inhibitor
**-85.06**	ampicillin	Bacterial cell wall synthesis inhibitor
**-84.76**	rucaparib	PARP inhibitor
**-82.88**	CAY-10618	NAMPT inhibitor
**-82.43**	latrepirdine	Glutamate receptor antagonist
**-79.71**	GSK-1070916	Aurora kinase inhibitor
**-79.01**	norcyclobenzaprine	Adrenergic receptor agonist, Serotonin receptor antagonist

**-76.35**	PIK-90	PI3K inhibitor
**-76.28**	TG-101348	FLT3 inhibitor, JAK inhibitor
**-73.87**	tracazolate	GABA receptor modulator
**-73.68**	OMDM-2	FAAH inhibitor
**-73.06**	GDC-0941	PI3K inhibitor
**-73.06**	RO-15-4513	GABA benzodiazepine site receptor inverse agonist
**-71.55**	iopanoic-acid	Radiopaque medium
**-69.66**	RHO-kinase-inhibitor-III[rockout]	Rho associated kinase inhibitor
**-69.05**	denbufylline	Phosphodiesterase inhibitor
**-68.05**	esmolol	Adrenergic receptor antagonist
**-67.64**	acetyl-geranyl-cysteine	Isoprenylated protein methylation inhibitor
**-66.71**	tivozanib	VEGFR inhibitor
**-65.88**	cytochalasin-b	Microtubule inhibitor
**-64.63**	epothilone	Microtubule inhibitor
**-64.56**	nicergoline	Adrenergic receptor antagonist
**-64.52**	XMD-1150	Leucine rich repeat kinase inhibitor
**-63.96**	brazilin	Nitric oxide production inhibitor
**-63.08**	olaparib	PARP inhibitor

**-63.01**	BMS-641988	Androgen receptor antagonist
**-62.32**	aminopentamide	Acetylcholine receptor antagonist
**-61.02**	tosyl-phenylalanyl-chloromethyl-ketone	Chymotrypsin inhibitor
**-61**	HLI-373	MDM inhibitor
**-60.73**	KU-0063794	MTOR inhibitor
